# Supplementary material for: Neoadjuvant Gastric Cancer Treatment and Associated Nutritional Critical Domains for the Optimization of Care Pathways: A Systematic Review
Source: Nutrients. 2023 May 9;15(10):2241. doi: 10.3390/nu15102241 (PMC10223831; doi:10.3390/nu15102241)
Supplement: Supplementary file 1 [file nutrients-15-02241-s001.zip › nutrients-2338802-supplementary.pdf]

File S1. Supplementary data: search strategy example

Cochrane Library search strategy (3rd November 2021)

1# MeSH descriptor: [Stomach Neoplasms] 3 tree(s) exploded
MeSH descriptor: [Neoadjuvant Therapy] this term only
#3 MeSH descriptor: [Preoperative Period] 1 tree(s) exploded
#4 #2 or #3
#5 MeSH descriptor: [Outpatients] 1 tree(s) exploded
#6 MeSH descriptor: [Ambulatory Care] explode all trees
#7 "outpatients":ti,ab,kw (Word variations have been searched)
#8 #5 or #6 or #7
#9 "nutrition* support" ti,ab,kw (Word variations have been searched)
#10 "oral nutrition* supplement*"
#11 "protein supplement*"
#12 "energy supplement*"
#13 "nutritional counseling"
#14 "dietary advice"
#15 "food fortification"
#16 "food enrichment"
#17 MeSH descriptor: [Nutritional Status] explode all trees
#18 MeSH descriptor: [Anthropometry] explode all trees
#19 MeSH descriptor: [Body Composition] explode all trees
#20 #9 or #10 or #11 or #12 or #13 or #14 or #15 or #16 or #17 or #18 or #19
#21 #1 and #4 and #8 and #20
#22 MeSH descriptor: [Letter] explode all trees
#23 MeSH descriptor: [Editorial] explode all trees
#24 MeSH descriptor: [Animals] explode all trees
#25 MeSH descriptor: [Child] explode all trees
#26 MeSH descriptor: [Pediatrics] explode all trees
#27 MeSH descriptor: [Pregnant Women] explode all trees
#28 MeSH descriptor: [Inpatients] focus
#29 MeSH descriptor: [Hospital] explode all trees
#30 "ward":ti,ab,kw (Word variations have been searched)
#31 MeSH descriptor: [Terminal Care] 2 tree(s) exploded
#32 MeSH descriptor: [Palliative Care] 2 tree(s) exploded
#33 letter or editorial or "case report" or animal or rodent* or child* or infant or infancy or p?diatric* or pregnant or ambulatory or inpatient* or "nursing home*" or "long term care" or palliative or "end of life" or "terminal care"
#34 #22 or #23 or #24 #25 #26 or #27 or #28 or #29 or #30 or #31 or #32 or #33
#35 #21 not #34

File S2.

File S2. Limitations of the included studies

Study	***Limitation***
Palmela et al Portugal 2017	Retrospective design, single-centre recruitment, and small sample size.<br>Lack of staging laparoscopy. Some of the patients included in the study might have already had peritoneal disease, and this may explain the notable percentage of patients with disease progression during NAC-<br>The use of different regimens of CT drugs according to patient characteristics.
Yamaoka et al Japan 2014	Retrospective study performed in a single institution. Relatively small size of the studied population warrants further studies.
Tan et al UK 2015	Study with limited power paving the way for further evaluations in larger samples. BSA-based dosing for CT.

Zhang et al<br>China<br>2021	Single-centre retrospective study, with deficient sample size and follow-up time. Cut-off values are in line with current guidelines for sarcopenia diagnosing, but cut-off points for VFA and SFA have not been established.<br>Hand-grip strength and usual gait speed should be measured in addition to muscle mass in further research.
Zhou et al<br>China<br>2020	Due to the lack of an established cut-off point for low skeletal muscle and adipose mass, the first-quartile values for low skeletal muscle and adipose mass were used; usage of this cut-off point, to define low skeletal muscle mass, has been increasingly considered a good threshold indicator. Small retrospective study sample size warranting further investigation, to be further confirmed in prospective studies. Follow-up time was short requiring longer periods in future studies.
Zhang et al<br>China<br>2021	Retrospective single-centre study with a short follow-up time and small sample size. The number of patients with marked FAT loss was limited, which might have caused bias. The cut-off values for body composition changes during neoadjuvant treatment varied between the studies. Therefore, the optimal cut-off values for muscle mass loss, VFA loss, SFA loss and body weight loss were determined by X-tile plots. Some patients were not assessed for the pathologic response to neoadjuvant treatment; thus this factor was not included in the final model for survival.
Jiang et al<br>China<br>2021	Retrospective single-centre study with small sample size limits the ability to draw broader conclusions. The NAC is SOX, preventing further discussions with other CT option plans. Study included patients from one single academic medical centre, and therefore the outcomes may not be generalizable.
Rinninela et al<br>Italy<br>2021	Study with different cut-off to define muscle mass loss which thresholds depends on the characteristics of the studied population such as age, race and country. Secondly, most of the included studies were performed on Asian patients given the high prevalence of gastric cancer in Asia. Although we included data from the only available European studies, more research is needed to confirm our findings in non-Asian populations with gastric cancer.
Jin et al<br>China<br>2021	Retrospective single-centre study with small sample size limits the ability to draw broader conclusions. ROC curve for the pre-treatment CONUT score cut-off value was associated with a poor sensitivity.
Li et al<br>China<br>2020	Patients included in the analysis might have different plan treatments, such as in nutrition support, CT plans and CT cycles, which could affect immunity and nutritional status. Failing to incorporate this information might have biased results. Length of follow-up is relatively short and therefore long-term survival cannot be assessed. The nomogram developed has not been validated internally or externally, which would cast doubts on its generalizability.
Sun et al<br>China<br>2016	Retrospective single-centre study.
Zhao et al<br>China<br>2018	The effect of ERAS programs on patients who received NAC was not observed; differences between NAC patients and those without-NAC may be more significant than what was explored in this study. Single-centre clinical trial, and results from other centres are required. Long-term survival rate was not determined and, therefore, a follow-up to evaluate whether NAC in ERAS programs are benefit on the long-term survival is necessary.
Claudino *et al.*<br>Brazil<br>2019	Lack of comprehensive data on dietary intake, given the nature of the retrospective study.

Legend: NAC – neoadjuvant chemotherapy; CT – chemotherapy; VFA- visceral fat adiposity; SFA - subcutaneous fat adiposity; FAT – fat adipose tissue; SOX - S-1 and oxaliplatin; CONUT – Controlling Nutritional status
